# Supplementary material for: Caring for Canines: A Survey of Dog Ownership and Parasite Control Practices in Ireland
Source: Vet Sci. 2023 Jan 24;10(2):90. doi: 10.3390/vetsci10020090 (PMC9960321; doi:10.3390/vetsci10020090)
Supplement: Supplementary file 1 [file vetsci-10-00090-s001.zip › vetsci-2149526-supplementary.pdf]

Supplementary Material

Table S1. Full Questionnaire.

Section	Topic	Questions asked
1	Owner Demography	Q1. What is your gender?
		Q2. What is your age?
		Q3. How many dogs are in your household?
2	Dog Demography	Q4. What is your dog's name?
		Q5. What age is your dog?
		Q6. What breed is your dog?
		Q7. When did you get your dog?
		Q8. Where did you get your dog?
		Q9. Is this your first dog?
		Q10. Is your dog neutered?
		Q11. Gender of your dog.
		Q12. Has your dog ever had puppies?
3	Dog Health	Q13. How often do you bring your dog to the vet?
		Q14. Do you know what a zoonotic infection is?
		Q15. Has your dog ever had worms?
		Q16. What deworming product do you use?
		Q17. How many times do you deworm your dog? Q18. How many times have you dewormed your puppy?
		Q19. How many times have you dewormed your dog during her pregnancy?
		Q20. Why do you deworm your dog(s)?
4	Household	Q21. Do you have young children (0-8 years) living in your home?
		Q22. Do you wash your hands after touching your dog(s)?
		Q23. What type of environment do you live in?
		Q24. Do you have an enclosed garden or outdoor area in your home?
5	Dog Feeding	Q25. How often do you feed your dog(s) per day?
		Q26. Please specify for each of your dog's how many times you feed them per day.
		Q27. What type of food do you feed your dog(s)?
6	Dog Outdoor Access	Q28. Does your dog(s) have access to an outdoor area or an enclosed space?
		Q29. Is your dog(s) supervised while outside in an enclosed space?
		Q30. Where does your dog(s) spend most of the day?
		Q31. Do any other animals occupy the same space?
		Q32. Please state the animals that do share your dog's space.
		Q33. Does your dog(s) bring prey items back home?
		Q34. Please state what prey items your dog brings home.
7	Dog Toileting	Q35. Where does your dog(s) go to the toilet most of the time?
		Q36. How is excrement (faeces/poo) disposed of?
8	Dog Walking	Q37. How many times a day is your dog(s) walked?
		Q38. Please specify how many times a day each dog is walked.
		Q39. What type of environment do you walk your dog(s) in most often?
		Q40. Is your dog(s) allowed off the lead?
		Q41. Does your dog(s) perform coprophagic behaviour while outdoors (eat faeces/poo)?
		Q42. Does your dog(s) perform scent rolling while outdoors (roll on the ground repeatedly back)?

Full summary table of the questionnaire results

**Variable**	**n**	**%**
**Participant gender**		
Male	137	20.6
Female	521	78.5
Other	6	0.9
**Participant age**		
18-24	239	36
25-34	113	17
35-44	121	18.2
45-54	116	17.5
55-64	54	8.1
65+	21	3.2
**Total dogs in household**		
1	513	77.3
2	121	18.2
3	20	3
4	10	1.5
**Dog age (note: $x \leq 0 - 6 \geq x$ ….)**		
< 6 months	12	1.4
6 months – 1 year	46	5.4
1 – 2 years	106	12.4
2– 3 years	85	9.9
3 – 4 years	67	7.8
4 – 5 years	64	7.5
5 – 6 years	67	7.8
6 – 7 years	57	6.7
7 – 8 years	48	5.6
8 – 9 years	54	6.3
9 – 10 years	42	4.9
10+ years	204	23.9
Don’t know	3	0.4
**Dog breed**		
Red setter/Gordon setter/Irish red setter/English setter/Red setter X	9	1.1
Golden retriever/Golden retriever X	49	5.7
Yorkshire Terrier/Yorkshire terrier X	45	5.3
**Jack Russell/Jack Russell terrier/Jack Russell X/Miniature Jack Russell**	**56**	**6.5**
German Shepherd/German Shepherd X	19	2.2
Chihuahua	24	2.8
Border collie/Collie/Border collie X/Collie X	43	5
Labrador/Labrador X/Labrador retriever	68	8
Cockapoo	44	5.1
Bichon Frise/Bichon frise X	36	4.2
Mongrel	14	1.6
Mixed/Mix/Mixed breed	20	2.3

Staffordshire Bull Terrier/Blue Staffordshire Bull Terrier	7	0.8
Pug/Puggle/Pugalier/Pug X	15	1.8
Pointer/English pointer/German pointer/ Miniature pointer/Wire hair pointer/Pointer X	14	1.6
Corgi/Pembrook Welsh Corgi/Corgi X	6	0.7
Cavachon	10	1.2
Sheepdog/Old English Sheepdog/Australian Sheepdog/Sheepdog X	6	0.7
Shih Tzu/Shih Tzu X	20	2.3
Greyhound/Sight hound	10	1.2
Springer Spaniel/Welsh/English springer spaniel/Springer spaniel X	31	3.6
Sato X	1	0.1
Cavalier King Charles/Cavalier X	26	3
Lurcher/Lurcher X/Whippet	20	2.3
Rottweiler/Rottweiler X	5	0.6
West Highland Terrier/Westie/Scottish terrier	8	0.9
Hungarian Vizsla	2	0.2
Schnauzer/Miniature schnauzer	11	1.3
Labradoodle/Mini labradoodle/Poodle	8	0.9
Tibetan terrier	1	0.1
Cavapoo	15	1.8
Pit Bull/Pit Bull X/American Pitbull Terrier	3	0.4
Spanish water dog	1	0.1
Weimaraner	2	0.2

**Zuchon**	**1**	**0.1**
Maltese/Maltese X	8	0.9
Terrier X/Cairn terrier/ Bedlington terrier/Norfolk terrier/Wirefox terrier/ Wheaten terrier/Glen of Imall terrier/ Patterdale terrier	36	4.2
Lhasa apso/Lhasa apso X	3	0.4
Dachshund/Dachshund X	13	1.5
Cocker Spaniel/English/Golden Cocker spaniel/Cocker spaniel X/Tibetan spaniel	28	3.3
Boxer/Boxer X	10	1.2
Finnish lapphund	1	0.1
Pomeranian/Pomeranian X	6	0.7
Beagle/Beagle X	6	0.7
Husky/Husky X/Siberian husky	10	1.2
Elkhound/Norweigan Elkhound	2	0.2
British/French Bulldog/Bulldog X	14	1.6
Greek shepherd Kokoni X	1	0.1
Japanese Spitz	4	0.5
Bernese Mountain dog/Bernese Mountain dog X	16	1.9
Rhodesian ridgeback	1	0.1
Great Dane	3	0.4
Hovawart	1	0.1

Papillion	3	0.4
Newfoundland/Newfoundland X	8	0.9
Belgium Malinois	2	0.2
Samojed	2	0.2
Glen of Imall terrier/Patterdale terrier	4	0.5
Portuguese Water Dog	2	0.2
Miniature Sausage Dog	1	0.1
Pekingese X	1	0.1
Alaskan Malamute X	1	0.1
Unknown	18	2.1
**Dog received**		
Pre pandemic	615	71.9
During pandemic	234	27.4
Unknown	6	0.7

**Where dog came from**		
**Family/Family friend**	**224**	**26.2**
Rescue/Shelter	205	24
Breeder	190	22.2
Farm	97	11.3
Online	47	5.5
Other	92	10.8
**First dog**		
Yes	259	39
No	405	61
**Neutered**		
Yes	494	74.4
No	170	25.6
**Sex**		
Male	439	51.3
Female	416	48.7
**Puppies**		
Yes	40	9.6
No	375	90.1
Unanswered	1	0.3
**Frequency of vet visits**		
Monthly	58	6.8
Every 3 months	143	16.7
Every 4 months	81	9.5
Every 5 months	36	4.2
Twice a year	244	28.5
Yearly	242	28.3
Never	46	5.4
Unknown	5	0.6
**Knowledge of zoonotic infections**		
Yes	205	30.9
No	459	69.1
**Past worm infection**		
Yes	198	23.2

No	652	76.3
Unanswered	5	0.5
**Deworming frequency (not including puppies or pregnant dogs)**		
**Monthly**	**73**	**9.4**
4-12 times a year	63	7.8
4 times a year	222	27.5
1-2 times a year	351	43.5
Never	88	10.9
Unanswered	5	0.6
**Deworming frequency for puppies**		
Weekly	0	0
Every 2 weeks	4	7.5
Twice a month	0	0
Monthly	21	39.6
Every 2 months	20	37.7
Never	7	13.2
Unanswered	1	2
**Deworming product used (participants also listed ectoparasite treatments, so these are included in the summary)** Administered/Prescribed by vet	95	11.1
Drontal/Drontal plus deworming tablets	47	5.5
Simparica trio	36	4.2
Troscan	34	4
Milbemax	25	2.9
Parazole liquid wormer	19	2.2
Milpro	17	2
Bravecto	17	2
Advocate	16	1.9
Gulliver's/Bob Martin Spot-on solution	13	1.5
Frontline	7	0.8
NexGard	9	1.1
Prowormer	8	0.9
Stronghold	5	0.6
Milprazon	4	0.5
Prinovox spot-on solution	2	0.2
Heart Guard	2	0.2
Prazitel plus	2	0.2
Beaphar liquid wormer	2	0.2
Panacur	2	0.2
Zantel	2	0.2
**Symposia**	**2**	**0.2**
Exitel	2	0.2
Interceptor plus	1	0.1
Dronspot spot-on wormer	1	0.1
Floradix	1	0.1
Vermox	1	0.1
Antihelmin plus	1	0.1

Cazitel	1	0.1
Triheart	1	0.1
None	9	1.1
Whatever is on sale	4	0.5
Don't know	145	17
Unanswered	324	37.9
**Reason for deworming dog (participants could choose all that applied)**		
Public health reasons	145	12.8
The vet told me to	283	24.9
To protect my dog	490	43.2
To protect myself	214	18.9
I do not deworm my dog	3	0.2
**Young children (0-8 years) in household**		
Yes	119	17.9
No	545	82.1
**Hand hygiene around dog**		
All the time	130	19.6
Sometimes	444	66.9
Never	90	13.5
**Environment**		
Urban	105	15.8
Sub-urban	348	52.4
Rural	211	31.8
**Enclosed garden/Outdoor area**		
Yes	624	94
No	40	6
**24 hr dog feeding frequency**		
Once	107	16.1
Twice	447	67.3
3 times	76	11.5
**4 times**	**2**	**0.3**
Other	22	3.3
Dogs are fed different amounts	10	1.5
**Type of food dog is fed (participants could choose all that applied)**		
Wet food	183	17.1
Dry food	627	58.5
Raw meat	31	2.9
Leftovers	177	16.5
Other	54	5
**Access to outdoors**		
Yes	651	98
No	13	2
**Supervised outdoors**		
Yes	320	42.2
No	344	51.8
**Where dog spends most of its time**		
Indoors	546	82.2

Enclosed back/front garden	90	13.6
Green area in an estate	3	0.4
Farm	16	2.4
Field	7	1.1
Unanswered	2	0.3
**Other animals**		
Yes	145	21.8
No	519	78.2
**Animals that share dogs' space**		
Cat(s)	78	49
Rabbits/Gerbils/Guinea pigs	9	5.7
Another dogs	20	12.6
Birds	14	8.8
Farm animals	26	16.4
Non-domestic wildlife	12	7.5
**Prey items**		
Yes	32	4.8
No	632	95.2
**Prey items brought home**		
Birds	9	28.1
**Birds and mice/rats**	**6**	**18.8**
Birds and cats	1	3.1
Birds, mice, and frogs	2	6.3
Birds, mice, and hedgehogs	1	3.1
Mice/rats	6	18.8
Mice/rats and cats	1	3.1
Rabbits/Hares	2	6.3
Rabbits and mice/rats	3	9.4
Unanswered	1	3.1
**Toileting location**		
Garden	391	58.9
Outdoors	151	22.7
On walk	75	11.3
Park	24	3.6
Field/Farm	16	2.4
Indoors	7	1.1
**Dog faeces disposal**		
General waste bin/Picked up and binned	411	61.9
Not disposed of	95	14.3
Compost bin	89	13.4
Sewer/Drain in garden	15	2.3
Dog bins	10	1.5
Flushed down toilet	10	1.5
Other	34	5.1
**24 hr dog walking frequency**		
Once	286	43.1
Twice	194	29.2
3 times	55	8.3

4 times	17	2.6
Dog not walked everyday	95	14.3
Dogs are walked at different times	17	2.6
**Environment dog walked in**		
Public Park	233	35.1
Street	190	28.6
Field or farmland	115	17.3
Dog Park	10	1.5
Park with children's play area	3	0.5
Other	113	17
**Dog allowed off the lead**		
Yes	395	59.5
No	269	40.5
**Coprophagy**		
Often	53	8
Rarely	186	28
Never	425	64
**Scent rolling**		
Often	198	29.8
Rarely	281	42.3
Never	185	27.9
